# Comparison of outcomes of arterial end-to-side versus end-to-end anastomosis in autologous deep inferior epigastric perforator (DIEP) flap breast reconstruction: DIEP-ES study protocol

**DOI:** 10.1097/SP9.0000000000000035

**Published:** 2025-12-21

**Authors:** Oliver Didzun, Adriana C. Panayi, Iman Ghanad, Sophie Osenegg, Laura Siegwart, Emre Gazyakan, Felix Vollbach, Ulrich Kneser, Amir K. Bigdeli

**Affiliations:** a Department of Hand, Plastic and Reconstructive Surgery, Burn Center, BG Center Ludwigshafen, University of Heidelberg, Ludwigshafen, Germany; b Department of Hand and Plastic and Surgery, Ruprecht-Karls-University Heidelberg, Heidelberg, Germany

**Keywords:** anastomosis, autologous breast reconstruction, breast reconstruction, DIEP flap, microsurgery

## Abstract

**Background:**

End-to-end anastomosis to the internal mammary artery (IMA) is the current standard anastomosis technique for women undergoing autologous breast reconstruction with deep inferior epigastric perforator (DIEP) flap. This approach fails to preserve the length of the IMA, compromising its availability for cardiac surgery use in women who develop coronary heart disease. A viable alternative may be end-to-side anastomosis, but data on its feasibility is currently lacking.

**Methods:**

This pilot study will involve 60 patients at a single-center institute over approximately 24 months. Inclusion criteria are female sex, age >18 years, history of breast cancer, and eligibility for unilateral autologous DIEP breast reconstruction. Exclusion criteria include patients with a legal guardian, inability to consent, and history of blood clotting disorders or hypercoagulability. Data will be collected at 2 weeks, 6 weeks, 6 months, and 1-year post-surgery. The primary outcome is “major complication” of the recipient site, including anastomotic insufficiency, arterial thrombosis, hematoma, reoperation, or flap loss. Secondary outcomes are abdominal perfusion and major donor site complications, such as wound healing disorders. Patient satisfaction will be assessed using the SF-36 and BREAST-Q (reconstructive module) questionnaires.

**Study status:**

The DIEP-ES study has begun enrolment in February 2023. As this is an ongoing trial, no results have been gathered yet. The results will be reported upon completion of the study. We anticipate that the primary and secondary outcomes of the end-to-side approach will be comparable to the accepted standard of care, i.e., end-to-end anastomoses.

**Conclusions:**

End-to-side anastomosis may be a safe alternative for DIEP breast reconstruction, especially for patients at risk for coronary heart disease. This pilot study aims to evaluate the feasibility and safety of the end-to-side anastomosis technique in DIEP-flap breast reconstruction. The preliminary findings will inform the design of future multicentric trials to confirm the efficacy of this approach.

## Introduction

According to the World Health Organization, breast cancer is currently the most prevalent cancer worldwide; in 2020 alone, 2.3 million women received a new diagnosis, while by the end of the same year, there were 7.8 million women who had been diagnosed with breast cancer since 2015. In terms of quality of life, more disability-adjusted life years are lost by women due to breast cancer than any other type of cancer globally.^[^1^]^ Previous studies outlined that breast reconstruction does not just improve patient quality of life and decrease stress and anxiety levels but also helps patients to better psychologically accept a breast cancer diagnosis.^[^2^,^^3^^]^ Therefore, it is not surprising that the proportion of women who are high risk, including women with comorbidities, women older than 60 years, and women with aggressive types of breast cancer, who opt to undergo breast reconstruction has increased more than threefold since the late 1990s.^[^4^]^

When immediate or two-stage implant-based breast reconstruction is not possible, is declined by the patient, or fails over time, free autologous breast reconstruction is recommended.^[^5^,^^6^^]^ In 2010, 28% of plastic surgeons in the United States reported a preference for performing breast reconstruction using the deep inferior epigastric perforator (DIEP) flap, making it the most important free flap in autologous breast reconstruction.^[^7^]^ The current surgical standard includes end-to-end anastomosis of the DIEP flap artery and the internal mammary artery (IMA) which, unavoidably, leads to complete transection of the distal end of the latter.^[^8^]^

As the makeup of the population changes, with projection studies predicting that future surgical cohorts will be older and more obese, it can be safely assumed that the rates of heart disease will also be higher.^[^9^]^ Furthermore, it is known that radiotherapy can increase the risk of ischemic heart disease, depending on the dose to the heart, particularly in women with preexisting cardiac risk factors.^[^10^,^^11^^]^ Despite a lack of high-level evidence on the proportion of patients who have undergone free autologous DIEP flap breast reconstruction and who developed ischemic heart disease, prior research has cautioned against complete transection of the IMA.^[^12-14^]^ Furthermore, there is evidence indicating that an arterial end-to-end anastomosis may compromise abdominal skin perfusion, thus potentially playing a role in the onset of abdominal wound healing disorders.^[^15^]^

Based on the currently available evidence, we hypothesize that the use of microsurgical end-to-side anastomosis in DIEP flap breast reconstruction surgery is a viable alternative to the standard end-to-end technique. We predict that the less commonly used end-to-side technique is not associated with an increased risk of flap loss, whilst being associated with lower abdominal complication rates. Simultaneously, it allows for the preservation of the IMA for future use as a bypass graft in coronary artery bypass surgery. The DIEP-ES study will be the first pilot study to compare arterial end-to-side and arterial end-to-end anastomosis in DIEP flap breast reconstruction surgery. Given the contradictory results in the literature on the surgical complication rates between arterial end-to-side and end-to-end anastomosis, a prospective pilot study looking at major complication rates is imperative for sample size calculation of large-scale multicenter trials.

## Methods

### Study setting

The DIEP-ES trial will be conducted in the BG Center Ludwigshafen - Department of Hand, Plastic and Reconstructive Surgery.

### Study participants

We include any patients planned for unilateral DIEP flap breast reconstruction who received prophylactic mastectomy or mastectomy due to breast cancer treatment, and who are aged 18 years or above. We exclude any patients with a legal guardian, lack of ability to communicate or capacity to provide consent, and patients with a history of a blood clotting disorder or thrombosis-promoting diseases. By including patients with a history of cancer, we ensure to include the main cohort of patients who tend to receive breast reconstruction. Furthermore, by excluding patients receiving bilateral DIEP flap reconstruction, abdominal skin perfusion is not biased by the type of reconstruction and can be assessed more objectively. Finally, we exclude all patients with blood clotting disorders or thrombosis-promoting secondary disease to avoid any predisposition to complication rates.

### Study design

**Figure 1.**
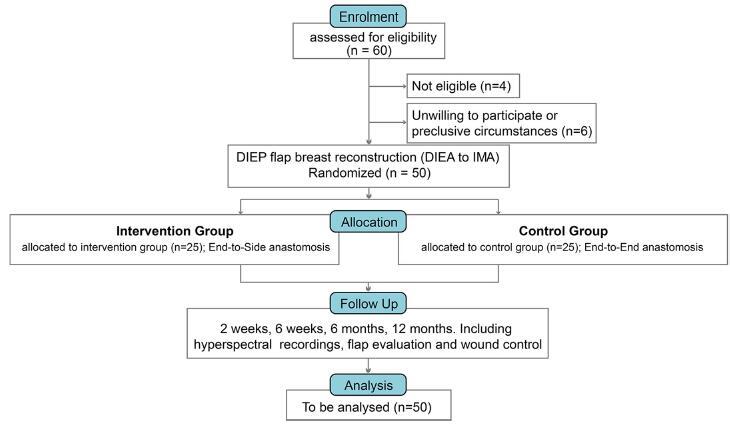
Study design. DIEP, deep inferior epigastric perforator; DIEA, deep inferior epigastric artery; IMA, internal mammary artery.

The study will follow a sequential design starting with patient recruitment. Patients will be screened based on inclusion and exclusion criteria, and those eligible will be randomized into either the end-to-side or end-to-end anastomosis group. The surgical procedure will be performed by experienced surgeons, and patients will be followed up at 2 weeks, 6 weeks, 6 months, and 12 months post-surgery. Data will be collected on primary and secondary outcomes. The study design is detailed in Fig. 1. The success of the surgical techniques will be evaluated using the following objective criteria: (1) Flap viability and complications, assessed through clinical examination, (2) perfusion at the donor-site, assessed using hyperspectral imaging, (3) patient satisfaction, measured using the SF-36 and BREAST-Q questionnaires at multiple time points post-surgery.

### Control(s)/comparator(s)

We chose arterial end-to-end anastomosis of the flap artery, namely the deep epigastric artery, and IMA as the control group since it is a more recently proposed surgical option in DIEP flap breast reconstruction.^[^10^]^ Transection of the IMA may not just impact abdominal skin perfusion, but also compromise future coronary artery bypass surgery, a major reason for the necessity of comparing the newly described arterial end-to-side anastomosis with the well-established arterial end-to-end anastomosis.

### Primary outcomes

The primary outcome is “major complication” at the recipient site, defined as the occurrence of microsurgical complications (anastomotic insufficiency, arterial thrombosis), hematoma, seroma, reoperation, or partial or total flap loss. This was chosen as it is simple but objective, consistent, and easy-to-compare safety outcome.

### Secondary outcomes

The secondary outcomes of abdominal perfusion and major complications of the donor-site, which include abdominal wound healing disorders, were chosen based on the understudied association between transection of the IMA and compromised abdominal wound healing following DIEP flap breast reconstruction. By utilizing hyperspectral imaging to determine perfusion of the abdominal wall, as well as performing frequent clinical assessments of the donor sites’ wound healing, we aim, for the first time, to collect objective prospective data. Moreover, we included patient satisfaction, as evaluated through the SF-36 and the BREAST-Q (reconstructive module) questionnaires, as a secondary outcome. Since lack of satisfaction with the outcomes of mastectomy and implant-based breast reconstruction is one of the main reasons leading patients to seek autologous breast reconstruction, it is important to consider patient satisfaction and physical and psychological well-being.

### Recruitment

Relevant to the proposed project, 517 microsurgical procedures were performed, of which 60 were free autologous breast reconstructions. The BG Center Ludwigshafen holds weekly breast consultation clinics aimed at recruiting patients seeking autologous breast reconstruction. Furthermore, patients are selected at specialist consultation clinic appointments. Our selected recruitment number of 60 patients is well within the number of DIEP procedures performed annually at the BG Center Ludwigshafen and is realistically achievable.

### Treatments/procedures

During the screening visit, written informed consent and patient eligibility will be established. Furthermore, demographic, as well as health-related data, will be collected, and a standardized clinical examination will be performed.

During the baseline visit, laboratory blood tests and a second clinical examination will be performed. Hyperspectral imaging of the abdominal wall will be conducted using the Tivita® tissue camera system, (Diaspective Vision, Am Salzhaff, Germany) to define the baseline abdominal perfusion measurement for each patient. Hyperspectral imaging has been validated in previous research for its accuracy in assessing skin perfusion and related parameters. Lu and Fei reviewed the application of HSI in medical fields and highlighted its ability to capture spatially-resolved spectral data that provides quantitative information on tissue physiology and morphology. In particular, HSI has been successfully utilized to monitor skin conditions, detect ischemic areas, and evaluate tissue oxygenation and blood flow^[^16^]^. To assess the patients’ preoperative health-related quality of life and satisfaction, patients will be asked to fill out the SF-36 and BREAST-Q (reconstructive module) questionnaires. The SF-36 Health Survey is a generic instrument for the assessment of the patient’s health-related quality of life, while the BREAST-Q is a validated questionnaire to measure patient perception before and after breast reconstruction by examining various quality of life domains (psychosocial, physical, and sexual well‐being) and satisfaction domains (satisfaction with breasts, and with outcome).^[^17^,^^18^^]^ Furthermore, preoperative computed tomography angiography (CTA) of the abdominal wall ensures adequate planning for intraoperative perforator dissection.

Post-surgery, hourly clinical flap perfusion controls will be performed and documented for five days. Furthermore, the Tivita® Tissue apparatus will be used to take hyperspectral images to measure flap and donor-site oxyhemoglobin level (Oxy), deoxyhemoglobin level (Deoxy), and oxygen saturation (O_2_Sat). More specifically, the device will be held at a standard, predefined distance from the site of interest, and tissue scan images will be obtained. The images will be electronically stored and analyzed using the company’s algorithm. Patients will be followed up on days 1, 3, and 6, as well at 6 weeks, 6 months, and 12 months with hyperspectral imaging and clinical examination, after 12 months, the SF-36 and BREAST-Q questionnaires will be filled out again.

### Surgical procedure

In all patients, the internal mammary vessels will be rapidly exposed, typically at the 3rd or 4th intercostal space. Following the splitting of the pectoralis major muscle along its fibers and its subsequent retraction, the intercostal muscle layer will be divided using a bipolar electrocautery device. If deemed necessary, the medial portion of the rib cartilage will be removed. Under loupe magnification, the internal mammary vessels will be carefully dissected, and any side branches present will be clipped and divided. After positioning the flap, venous anastomoses will be executed using venous coupler devices, whereas all arterial anastomoses will be performed manually with 8-0 nylon sutures under a microscope.

For end-to-side arterial anastomosis, microvascular clamps will be applied, and an arteriotomy will be created by making an elliptical excision of the arterial wall using scissors. Suturing will begin at the 6-o’clock position (defined as the caudal edge of the excision) and proceed sequentially through the 5-o’clock, 7-o’clock, 4-o’clock, 8-o’clock, 3-o’clock, 9-o’clock, 2-o’clock, 10-o’clock, 1-o’clock, 11-o’clock, and 12-o’clock positions. This approach ensures constant visibility of the arterial lumen, preventing inadvertent fixation of the opposite vascular wall. In end-to-end arterial anastomosis, the IMA will be transected using scissors. Following the transection, suturing will commence at the 6-o’clock position and proceed sequentially as described for the end-to-side anastomosis.

### Frequency and scope of trial visits

**Figure 2.**
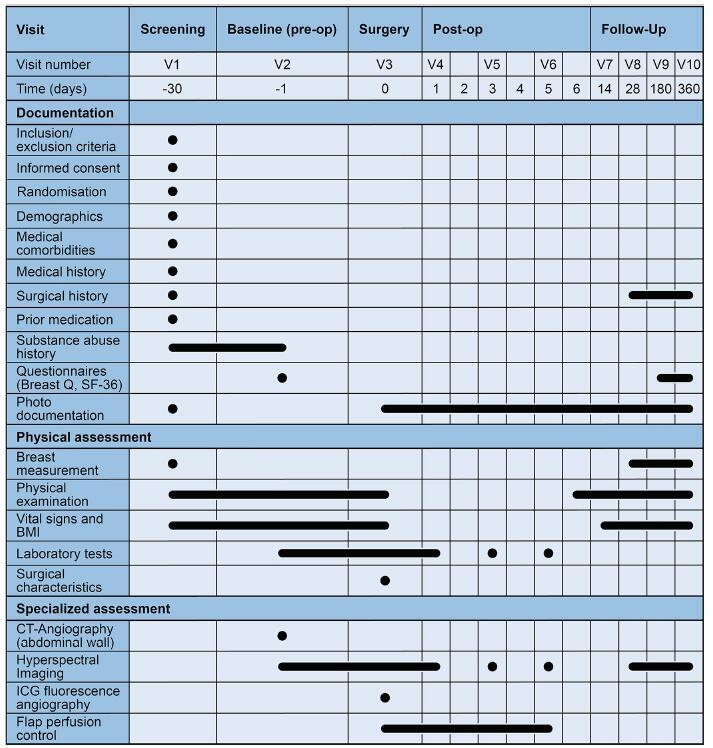
Frequency and scope of trial visits.

A schedule of trial enrolment, interventions, and outcome measurements for each individual patient is presented in Fig. 2. The study’s total duration is anticipated to span 24 months, from first-patient-in (FPI) to last-patient-out (LPO).

### Serious adverse event reporting

Serious adverse events, not related to the study treatment, are any complications that result in the following: a life-threatening situation, requirement of transfer to an internal medicine department, death, or permanent or significant disability. Patients who experience such adverse events will be excluded from the final analysis. Any serious adverse events will be reported to the ethics committee that approved the protocol, within 7 days of the occurrence of the event.

### Patient involvement

No patients were involved in the conceptualization of the study, the proposal of the research questions, the design of the study, or patient recruitment.

### Methods against bias

To avoid inadvertent selection bias, we will prospectively assign patients to the intervention or control group based on a purely random fashion (i.e., fair coin toss).

Although no patients will be excluded based on race or other demographic characteristics, the mono-centric nature of this study predisposes to confounding bias. Specifically, patients treated at our institution tend to have geographical proximity and are usually of European and white descent. This is in line with the demographics of Germany.^[^19^]^ This confounding bias is, however, inherently associated with monocentric studies and can only be avoided with future multicenter studies that investigate larger populations and can help compensate for such bias by offering greater geographic, socioeconomic, and peri-operative variability.

As is frequently the case with trials involving surgical procedures, neither surgeons nor patients will be blinded to the procedure part of the study (i.e., which group the patient is placed in): in the case of surgeons this is because of obvious reasons, and in the case of patients this is because we will seek to provide the patients with complete and transparent information on any future cardiac intervention therapeutic options. Awareness that the trial is offering patients the best treatment available has previously been identified as a major motivation factor for patients’ agreement to a clinical trial.^[^20^]^ However, to limit such bias as much as possible, the evaluators of the post-treatment outcomes, for example, those assessing the hyperspectral images and satisfaction surveys, will follow a standardized blinded regimen. This standardized assessment regimen will be performed according to a strict protocol that is designed to limit any detection bias that classically occurs when the assessment methods of the outcomes are not consistent between the experimental and control group. The use of standardized outcome measures, for example, the BREAST-Q (reconstructive module) and SF-36 questionnaires, will help limit bias arising due to the subjective nature of postoperative satisfaction.

### Power calculations

Given the nature of this trial (i.e., pilot study) the primary goal is confidence interval (CI) estimation for future studies, rather than formal sample size calculation. Regardless, pilot studies are used to inform sampling decisions for future larger studies. As such, biometric consultation was sought from the Institute of Medical Biometry and Informatics (IMBI), Heidelberg University. Our sample size determination is based on empirical values derived from previous surgical studies, suggesting that a sample size of 60 patients is pragmatic and sufficient for data collection within a feasible timeframe. This approach is supported by guidelines from Lancaster, Dodd, and Williamson, recommending an overall sample size of 30 per group for feasibility and pilot studies with quantitative end goals^[^21^]^. Given a complication rate – as reported by Kim *et al* – of 6% and 10% for end-to-side and end-to-end anastomosis, respectively, the expected confidence interval range is 35.8%^[^22^]^. Assuming a complication rate of 10% in both patient groups, the calculated confidence interval range is 37.6%. Based on the feasibility-study-character of this trial with primarily binary endpoints, such confidence intervals are acceptable. With an estimated enrollment of one patient per week, we expect a total recruitment period of slightly over 12 months. Taking into consideration the 12-month follow-up period, the total duration of this study is estimated to be approximately 24 months, however, the study will continue until the total number of 60 patients enrolled is reached.

### Ethical considerations

The protocol has been written, and the study will be conducted, according to the ICH Harmonized Tripartite Guideline for Good Clinical Practice (ref: https://www.ema.europa.eu/en/documents/scientific-guideline/ich-e-6-r2-guideline-good-clinical-practice-step-5_en.pdf). The DIEP-ES trial has been approved by the local ethic committee of (anonymized data) and registered at the German Clinical Trials Register.

### Data monitoring

A pre-selected independent data monitoring committee comprising qualified international experts will monitor compliance with the study protocol and the collected data’s quality.

### Confidentiality and data storage

All data will be stored in a de-identified format. In addition, no identifying information will be used in any publications resulting from this study. Data will be stored on a local server of the Department of Hand, Plastic and Reconstructive Surgery, Burn Center, BG Center Ludwigshafen.

### Information dissemination

The results of this study will be submitted for publication to international high-impact, peer-reviewed, scientific journals, regardless of outcome. The results will also be presented at international conferences.

### Study status

Enrollment has begun on February 1, with data collection projected to conclude by 1 February 2025. As the trial is currently ongoing, no results have been collected to date. The study’s findings will be reported upon the trial’s completion. We anticipate that the primary and secondary outcomes for the end-to-side approach will be comparable to the established standard of care, specifically the end-to-end anastomoses.

## Discussion

In 2015, a long-term follow-up of an autologous breast reconstruction cohort by Fortin *et al* identified that 2.5% of the patients had developed ischemic heart disease that necessitated therapeutic management. Contrasting this to the lower rate of flap loss (1.7%) underscores the need for correct and appropriate selection of the anastomosis technique with the recipient vessel to pre-emptively optimize cardiac care for patients who develop coronary heart disease.^[^13^]^

The left IMA is the most important donor vessel in coronary artery bypass surgery. Its use is associated with significantly improved morbidity and mortality compared to other arterial or venous grafts.^[^22-24^]^ End-to-end anastomoses in autologous DIEP breast reconstruction, which has long served as the standard, does not allow for the preservation of the length of the IMA in the majority of cases, meaning that the vessel no longer suffices for future coronary bypass surgery.^[^14^]^ In contrast, end-to-side anastomosis, which can be performed at any height, can be planned in such a way that pre-emptively spares the internal mammary vessels for future use. Although high-quality rigorous studies are currently lacking, prior retrospective research has recommended the selection of end-to-side anastomosis in patients identified to have a higher cardiovascular risk.^[^25^]^ As such, end-to-side anastomosis in DIEP flap breast reconstruction can be planned in such a way as to preserve the internal mammary vessels for possible future cardiac surgery use, and, hence, ultimately reduce morbidity and mortality.

This assumption is supported by the work of Rozen *et al*, who did not identify any fundamental contraindication for coronary bypass surgery after DIEP flap breast reconstruction.^[^14^,^^26^^]^ In 2011, Apostolides *et al* conducted a retrospective study using data from 22 patients to compare end-to-end and end-to-side anastomosis in DIEP flap breast reconstruction surgery for the first time. No significant differences were observed, except for a moderate prolongation of ischemia time after flap elevation using end-to-side anastomoses.^[^27^]^ Based on these results, the authors concluded that using end-to-side anastomosis in DIEP flap breast reconstruction surgery is a reliable and technically feasible method. The results of Kim *et al* in 2012 supported these conclusions.^[^28^]^ Furthermore, Kim *et al* noted that the occurrence of postoperative complications was halved with end-to-side anastomoses, albeit, not significantly. Despite the retrospective study design and a relatively small number of patients, these results seem plausible given that end-to-side arterial anastomosis be as reliable as end-to-end anastomosis.^[^29^,^^30^^]^ It should also be noted that prior research has identified a correlation between abdominal wound healing complications and choice of anastomoses. Specifically, in 2017, Nergård *et al* proposed that end-to-end anastomosis may significantly impact abdominal skin perfusion and could be a driver for abdominal wound healing disorders, which at 13.7% represent one of the most common complications after DIEP-flap breast reconstruction.^[^15^]^

The DIEP-ES pilot study aims to prospectively collect reliable data to compare the intra- and postoperative complications and outcomes, including flap loss rates, abdominal perfusion, patient satisfaction, and perception of illness, in patients undergoing DIEP-flap breast reconstruction surgery either with end-to-side or end-to-end anastomosis. Data on abdominal perfusion and patient satisfaction has not been previously compared in the literature. If successful, this technique could preserve the IMA for future coronary artery bypass grafting, potentially reducing morbidity and mortality in patients with coronary artery disease. The data obtained will serve as the basis to (i) further standardize and improve the surgical procedure of reconstructive breast surgery and (ii) plan a confirmatory multi-center study.

### Limitations

As a pilot study, the sample size is small and may not be representative of the larger population. The monocentric nature of the study may introduce selection bias, and the lack of blinding could affect the subjective assessments of patient satisfaction. Future multicentric studies with larger sample sizes will be necessary to validate these findings.

## Data Availability

The data that support the findings of this study are available on request from the corresponding author [A.K.B.].

## Research registration unique identifying number (UIN)

DRKS, DRKS00032948 (Registered 27 October 2023 – retrospectively registered, https://drks.de/search/de/trial/DRKS00032948).
